# Genome-Wide Identification and Characterization of the Trihelix Transcription Factor Family in *Pinus massoniana* and Gene Expression Patterns Analysis

**DOI:** 10.3390/plants14233635

**Published:** 2025-11-28

**Authors:** Pengzhou Liu, Dengbao Wang, Shaojun Xu, Shuo Sun, Manli Yang, Meijing Chen, Kongshu Ji

**Affiliations:** 1 State Key Laboratory of Tree Genetics and Breeding, Nanjing Forestry University, Nanjing 210037, China; lpz9527@njfu.edu.cn (P.L.);; 2 Key Open Laboratory of Forest Genetics and Gene Engineering of National Forestry & Grassland Administration, Nanjing 210037, China; 3 Co-Innovation Center for Sustainable Forestry in Southern China, Nanjing Forestry University, Nanjing 210037, China

**Keywords:** *Pinus massoniana*, trihelix transcription factor, expression patterns

## Abstract

*Pinus massoniana* Lamb. possesses considerable ecological and economic value. However, the rapid proliferation of pine wilt disease poses a significant threat to the growth and development of coniferous plants. Transcription factors play a crucial role in enabling plants to respond to external environmental stresses. The trihelix transcription factor (TTF) family, named after its unique trihelical domain (helix-loop-helix-loop-helix) and also referred to as the GT family, plays crucial roles in plant morphogenesis and in responses to biotic and abiotic stresses. In this study, we identified 56 *PmGTs* from the *P. massoniana* genome and analyzed their expression profiles in response to pine wood nematode (PWN) infection. Eight significantly differentially expressed *PmGTs* at various stages were selected as candidate genes for PWN resistance. Promoter analysis and qRT-PCR revealed that these genes respond to multiple treatments, including methyl jasmonate (MeJA), indole-3-acetic acid (IAA), gibberellic acid (GA3), salicylic acid (SA), and abscisic acid (ABA). Subcellular localization analysis revealed that the proteins are localized in the nucleus. Additionally, seven *PmGTs* exhibit transcriptional activity. This study provides a foundational understanding of the role of *PmGTs* in stress response in *P. massoniana*.

## 1. Introduction

*Pinus massoniana* Lamb. exhibits rapid growth and possesses strong adaptability to adverse environmental conditions. making it a key pioneer species for afforestation in southern China and one of the country’s most important resin-producing trees [1]. China is the world’s largest producer of pine resin, exporting over half of its global supply. *P. massoniana* is the primary species responsible for resin production in China [2]. However, pine wood nematode (PWN) disease is currently regarded as the most destructive forest disease worldwide due to its rapid spread and the lack of effective prevention and control measures [3]. The disease is associated with an extremely high mortality rate, making it difficult to manage [4]. Consequently, PWN disease has been colloquially termed “pine tree cancer.” It has caused direct economic losses amounting to hundreds of billions of dollars, while the assessment of indirect economic losses and ecological damage remains challenging [5]. Therefore, elucidating the transcriptional response mechanisms to PWN infection in *P. massoniana* is crucial for the development of high-yielding and disease-resistant varieties.

Trihelix transcription factors (TTFs) contain a conserved triple helix-loop-helix-loop-helix domain, which enables them to specifically bind GT elements involved in light response regulation [6]. As a result, this gene family is also known as the GT factor family. First discovered in plants [7], GT factors have since been identified across a wide range of species. The amino acid sequences of the functional domains within GTs exhibit high sequence consistency and significant evolutionary conservation, typically localizing to the N- or C-terminal regions as one or two domains (Table 1). Based on the number of conserved amino acids in both the GT protein domain and the DNA binding domain, the GT family proteins are classified into five subfamilies: GT-1, GT-2, SH4, GTγ, and SIP1 [8].

Initial understanding of GT transcription factors (TFs) was primarily confined to their regulation of light-dependent target genes. As research has advanced, it has become evident that GT TFs play critical roles in diverse biological processes, including light signaling, leaf and flower development, and responses to both abiotic and biotic stresses [9,10,11,12,13,14,15,16,17]. Early studies showed that GT-1 binds to a fungal elicitor-responsive element in the PR-1a promoter, thereby repressing transcription. This finding initiated functional investigations into the role of GT-1 subfamily members in disease resistance. In *Arabidopsis thaliana*, the GT TF GTL1 functions as a downstream component of the MPK4 cascade, promoting salicylic acid biosynthesis and modulating bacterial-triggered immunity. Furthermore, *AtGT-3b*, *VFP3*, and *VFP5* act as positive regulators induced by *Pseudomonas syringae* and *Agrobacterium* infection, respectively, thereby enhancing plant resistance to pathogens. In contrast, the SH4 subfamily member ASR3 functions as a negative regulator during infection by highly virulent pathogens, attenuating disease resistance [18,19,20,21]. In *Oryza sativa*, expression of the GT-1 subfamily gene *OsRML1* is upregulated in seedling leaves upon infection with *Magnaporthe oryzae* [22]. Knockout of *ZmGT-3b*, a GT-1 subfamily member in *Zea mays*, results in upregulation of defense-related genes and enhanced pathogen resistance [23]. Overexpression of *GhGT-36_A04* in *Gossypium hirsutum* enhances responsiveness to *Verticillium dahliae* infection, resulting in significantly higher levels of reactive oxygen species (ROS) and callose deposition compared to wild-type plants. This effect is mediated through coordinated regulation of defense pathway genes, ultimately improving disease resistance [24]. In *Fragaria vesca*, GT TFs contribute to positive regulation under biotic stress by responding to pathogen attack and activating the salicylic acid (SA) and jasmonic acid (JA) signaling pathways [25]. Upon pathogen infection in *Actinidia deliciosa*, GT1 is upregulated, activating defense-associated genes such as PR1 and RIN4 and triggering ROS bursts, thereby strengthening immune responses against *Pseudomonas syringae* pv. *actinidiae* [26]. GT genes are widely recognized as positive regulators in defense responses. For example, in *Betula platyphylla*, GT-2 subfamily members-*BpTrihelix3*, *BpTrihelix4*, and *BpTrihelix7* are significantly induced by *Alternaria alternata* or *Rhizoctonia solani* due to the presence of disease resistance-related cis-regulatory elements [27]. In *Populus trichocarpa*, most Trihelix family members are upregulated after *Alternaria alternata* infection, although three members show downregulation. Suppression of *PtrGT10* in *Pyrus ussuriensis* reduces accumulation of reactive oxygen species (ROS) and malondialdehyde (MDA), along with decreased cell death, indicating its role in promoting defense-associated programmed cell death [28]. Collectively, GT TFs exhibit functional versatility in regulating plant immunity through bidirectional modulation of immune signaling networks and key defense gene expression. On one hand, they integrate MAPK signaling to promote ROS and callose accumulation or activate SA/JA pathway genes, thereby reinforcing basal resistance. On the other hand, certain members negatively regulate immunity by binding to promoters of immune genes or directly repressing defense gene expression. Therefore, systematic identification and characterization of GT family members in *P. massoniana* may provide valuable candidate genes for screening key regulators involved in resistance to pine wood nematode disease.

In this study, we identified 56 *PmGT* genes in *P. massoniana* and conducted a comprehensive analysis of their phylogenetic relationships, sequence characteristics, conserved domains, and subcellular localization. We further investigated the expression profiles of eight candidate genes across multiple tissues and under various abiotic stress conditions. Notably, we systematically characterized the transcriptional responses of *PmGT* genes to PWN infection at five distinct time points using RNA-seq data derived from resistant *P. massoniana* individuals. This study provides valuable insights into the potential role of PmGT genes in mediating resistance to PWN, contributing to the sustainable management of this economically important conifer species.

## 2. Results

### 2.1. Identification of GT Genes in P. massoniana

After conservative domain prediction, multiple sequence alignment, and removal of duplicate sequences, a total of 56 GT genes were identified and named *PmGT1* to 56 based on their chromosomal localization (Table S1). The protein sequences are available in Supplementary Materials Table S2. Among the 56 PmGT proteins, the number of amino acids was between 117 and 971, with the predicted molecular weight ranging from 13.61 to 107.19 kDa. The pI values ranged from 4.69 to 10.88. The detailed range of protein molecular weights and the isoelectric point values are listed in Table S1. Instability index and grand average of hydropathicity (GRAVY) results showed that *PmGT25*, *PmGT44* and *PmGT46* are stable proteins, whereas the remaining members are unstable. In addition, all members are hydrophilic proteins. According to the subcellular localization predications from CELLO, and Wolf PSORT, almost all GT genes were predicted to be localized at nucleus, with only *PmGT8* localized at chloroplast.

### 2.2. Chromosomal Distribution of GT Genes

**Figure 1:**
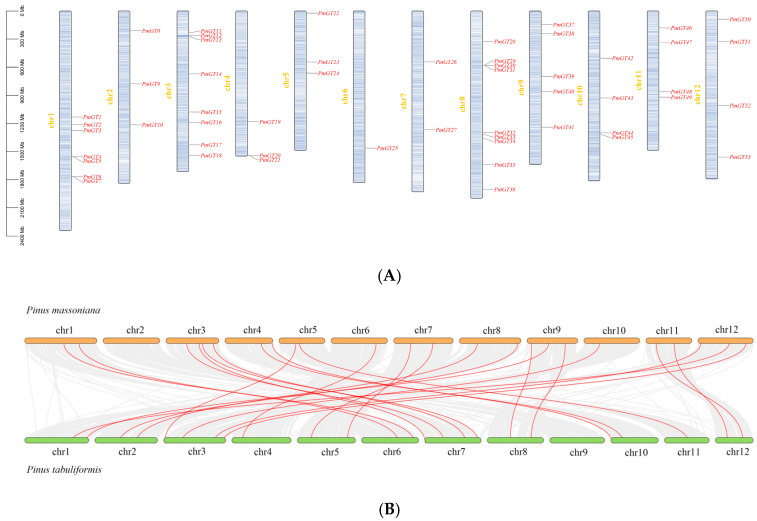
(**A**) Locations of *PmGTs* on *P. massoniana* chromosomes. (**B**) Synteny analysis of GTs between *P. massoniana* and *P. tabuliformis*.

The positions of *PmGT* genes were obtained from the genome annotation files. The 56 *PmGT* genes exhibited an uneven distribution across 12 chromosomes. Notably, chromosomes 6 contained only one *PmGT* gene, while chromosome 8 harbored nine Pmtrihelices (Figure 1A). Followed by chr3 and chr1 (8 and 7, respectively). *PmGTs* formed 25 syntenic gene pairs with *P. tabuliformis*. Syntenic analysis provided indirect evidence for the potential functional conservation of GT genes between the two pine species (Figure 1B); gene pairs exhibiting higher sequence homology likely originated from a common ancestral gene.

### 2.3. Phylogenetic and Protein Domain Analysis of PmGT Proteins

**Figure 2:**
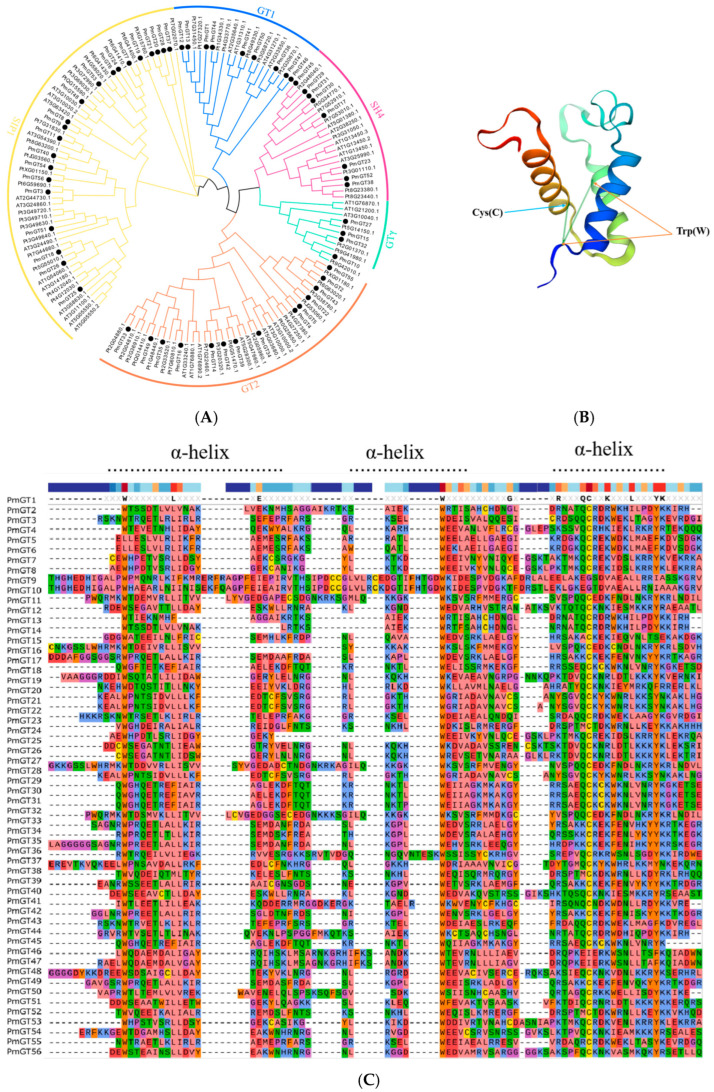

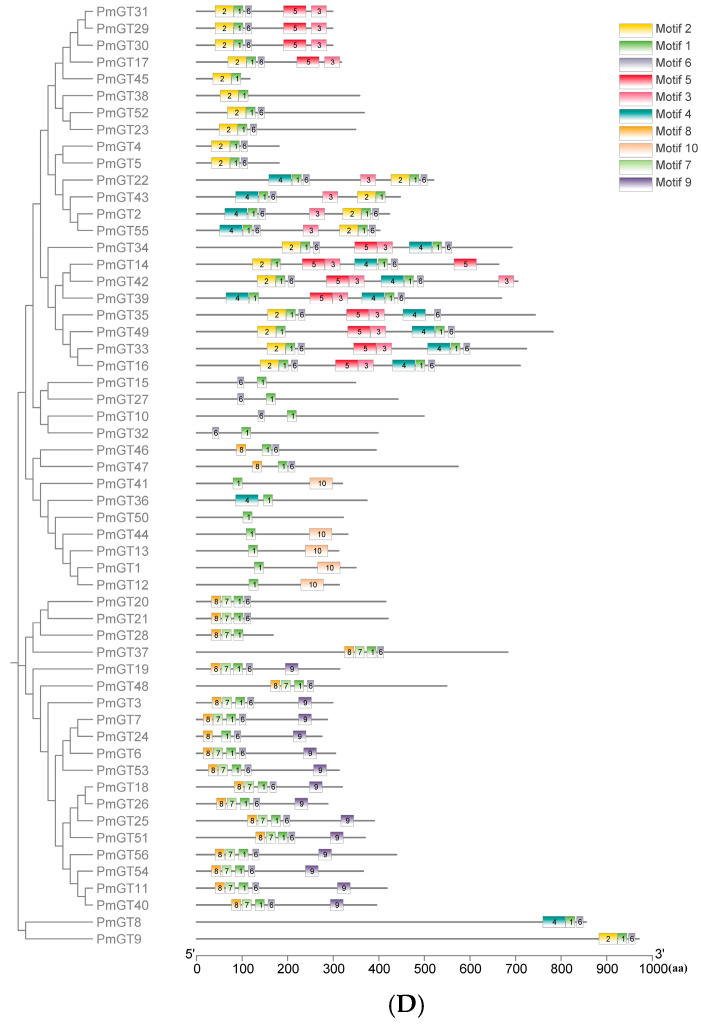
(**A**) Phylogenic tree of GT proteins in *P. massoniana*, *P. tabuliformis* and *A. thaliana*. Different colors represent different GT subfamilies. (**B**) The tertiary structure and highly conserved amino acids of trihelical domain in *P. massoniana*. (**C**) Multiple sequence alignment of GT protein family in *P. massoniana*. (**D**) Phylogenetic analysis and conserved motif distribution of *PmGT* genes.

Based on phylogenetic analysis in conjunction with *Arabidopsis thaliana* and *P. tabuliformis*, all *PmGTs* were divided into five subfamilies: GT-1, GT-2, GTγ, SIP1 and SH4. The group with the largest number was SIP1 (21 members), followed by GT-2 (14 members), then was GT-1 (9 members) and SH4 (8 members), and the smallest group was GTγ (4 members) (Figure 2A). Prediction protein 3D model and multiple sequence alignment showed that GT family contained a conserved triple helix-loop-helix-loop-helix domain (Figure 2B,C). According to MEME program identification (Figure 2D), we found that all members contained motif1. Interestingly, GT-2 subfamily exhibited the highest diversity of motifs. Motif 9 was only found in SIP1 subgroup. All motifs’ sequences were listed in Table 2. According to the phylogenetic tree, these conserved motifs in the same groups support the reliability of these group classifications.

### 2.4. Cis-Regulatory Elements Analysis of the PmGTs Promoters

**Figure 3:**
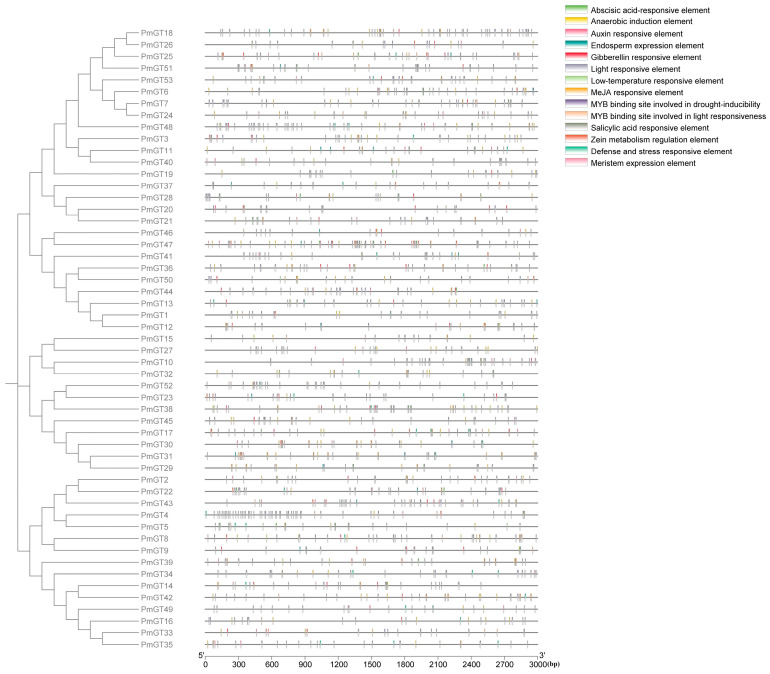
Analysis of cis-acting elements in the promoter of the *PmGT* gene family.

Cis-element analysis (Figure 3) showed the types and distribution of common regulatory elements in the promoters of *PmGT* genes. The result showed that these promoters contain a large number of light-responsive and phytohormone-related elements, including ARE (anaerobic induction), ABRE (ABA responsiveness), G-box (light responsiveness), TGACG-motif (MeJA responsiveness) and TCA (SA responsiveness), MYB, P-box (gibberellin responsiveness) and TGA-element (auxin responsiveness). Notably, all promoters collectively contained 891 light-responsive cis-acting elements, accounting for nearly 50% of total predicated cis-elements, indicating their important functional roles in the regulation of light-responsive processes. Other cis-element types were broadly distributed across all *PmGT* subfamilies: AREs constituted 12.0%, ABREs 7.6%, TGACG-motifs 7.4%, P-boxes 3.8%, TGA-elements 2.5%, and TCA-elements 1.7%.

### 2.5. Expression Patterns of PmGTs Under Nematode Stress

**Figure 4:**
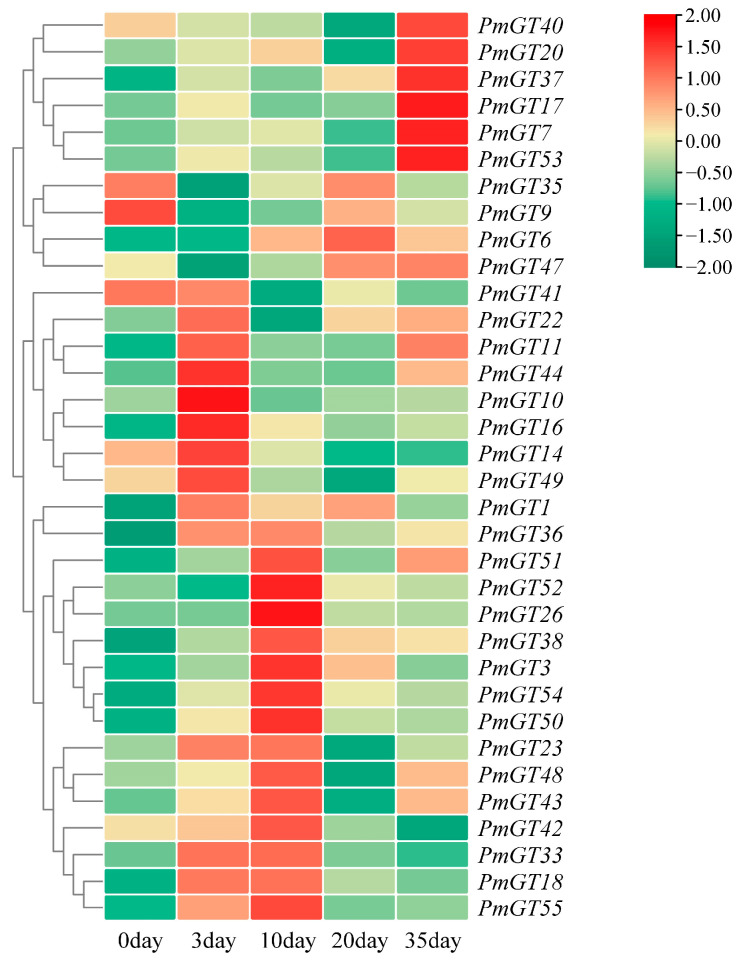
Transcriptional profiles of GTs in *P. massoniana* under nematode stress.

A heatmap was generated to analyze the expression levels of 34 *PmGTs* under nematode infestation based on the transcriptome data (Figure 4). After infection by nematodes, 24 *PmGTs* showed induced expression patterns at first 10 days while 6 members at 35 days. In addition, *PmGT6*, *PmGT9*, *PmGT35* and *PmGT47* were suppressed for the first 3 days, and then gradually rose, reaching the peak in 20 days. This early dip followed by sustained rise suggests a delayed but prolonged defense phase that coincides with the transition of *B. xylophilus* from migratory to propagative adults. According to expression patterns, we selected 8 members: *PmGT9*, *PmGT14*, *PmGT18*, *PmGT26*, *PmGT35*, *PmGT36*, *PmGT38* and *PmGT54*. These members exhibited significant changes in expression levels (q value < 0.05) between at least two stages and were chosen for further investigation.

### 2.6. Subcellular Localization Assay

**Figure 5:**
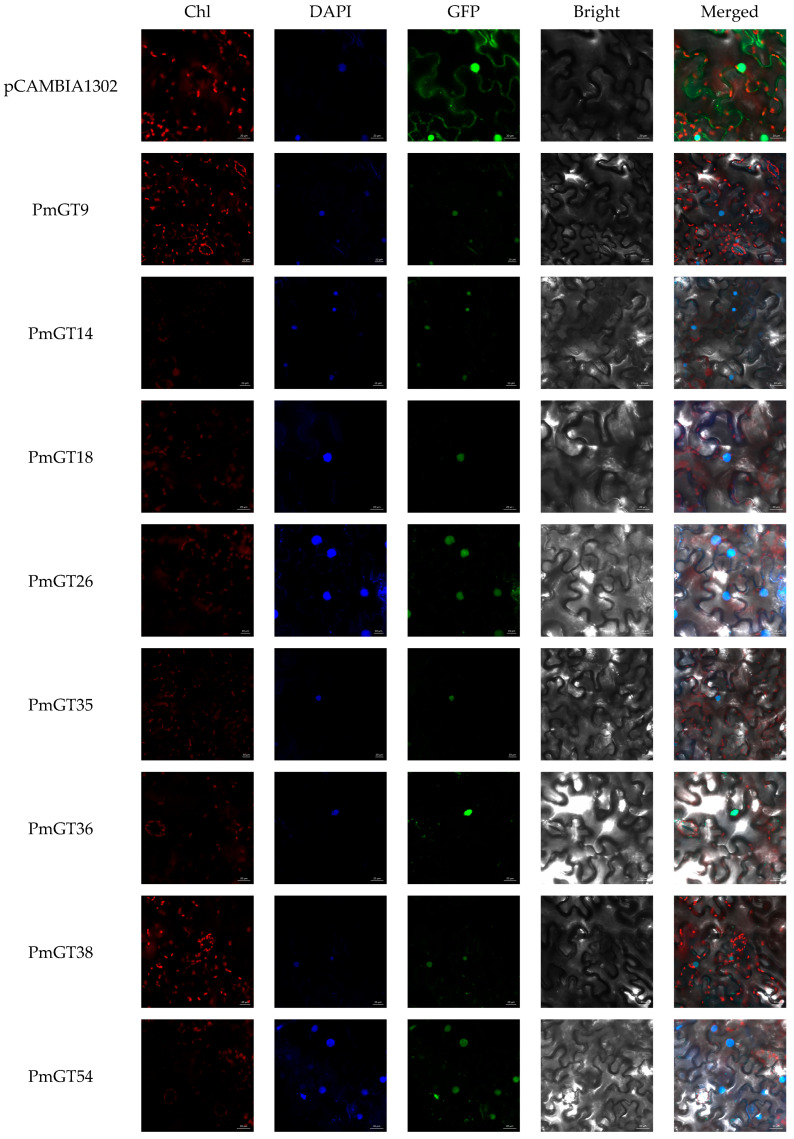
Subcellular localization of *PmGT* in epidermal leaf cells of transiently infected *N. benthamiana*. Bright: bright field. DAPI: 4′,6-diamidino-2-phenylindole, a blue fluorescent dye that shows DNA location. GFP: green fluorescence protein, displays the location of the target protein. Merged: merged picture of overlapped channels. Chl: chloroplast autofluorescence (far-red channel, 680–730 nm). Red is chloroplast auto-fluorescence. The scale in the images is 20 μm. pCAMBIA-1302-mGFP5 was control.

Subcellular localization prediction indicated that all selected *PmGTs* likely exert regulatory functions at the nucleus like most TFs. We used the empty vector pCAMBIA1302 as a negative control. Fluorescent signals were found after transient transformation in *Nicotiana tabacum* leaves. The green fluorescence signal was observed throughout the entire cell in the control, indicating the distribution of the green fluorescent protein. Red fluorescence represents chloroplast autofluorescence, while blue fluorescence marks the location of DNA, thereby revealing the position of the nucleus. However, mGFP5 fused with *PmGT9*, *PmGT14*, *PmGT18*, *PmGT26*, *PmGT35*, *PmGT36*, *PmGT38* and *PmGT54* exhibited fluorescence exclusively in the nucleus (Figure 5). These results indicate that all selected PmGT proteins are localized to the nucleus.

### 2.7. Expression Patterns in Different Tissues

**Figure 6:**
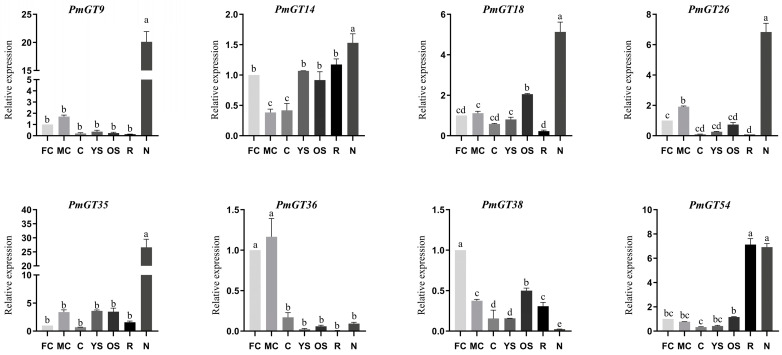
Expression patterns of *PmGTs* in different tissues in *P. massoniana*. FC: female cone, MC: male cone, C: cone, YS: young stem, OS: old stem, N: needle, R: root. Different letters in the same column indicate a significant difference, *p* < 0.05.

The qRT-PCR analysis in Figure 6 showed the expression patterns of eight *PmGTs* in seven different tissues: FC: female cone, MC: male cone, C: cone, YS: young stem, OS: old stem, N: needle, and R: root. It is evident that *PmGT9*, *PmGT14*, *PmGT18*, *PmGT26*, *PmGT35*, and *PmGT54* expressed mainly in needles. *PmGT54* expressed mainly also in root. *PmGT36* and *PmGT38* expressed mainly in female cone. The expression in *PmGT36* also detected highly in male cones. The significantly high expression levels in the needles suggest that these GT genes may play a key regulatory role in rapidly initiating local resin defense responses while perceiving mechanical damage or pathogen signals.

### 2.8. Expression Patterns Under Plant Hormones

**Figure 7:**
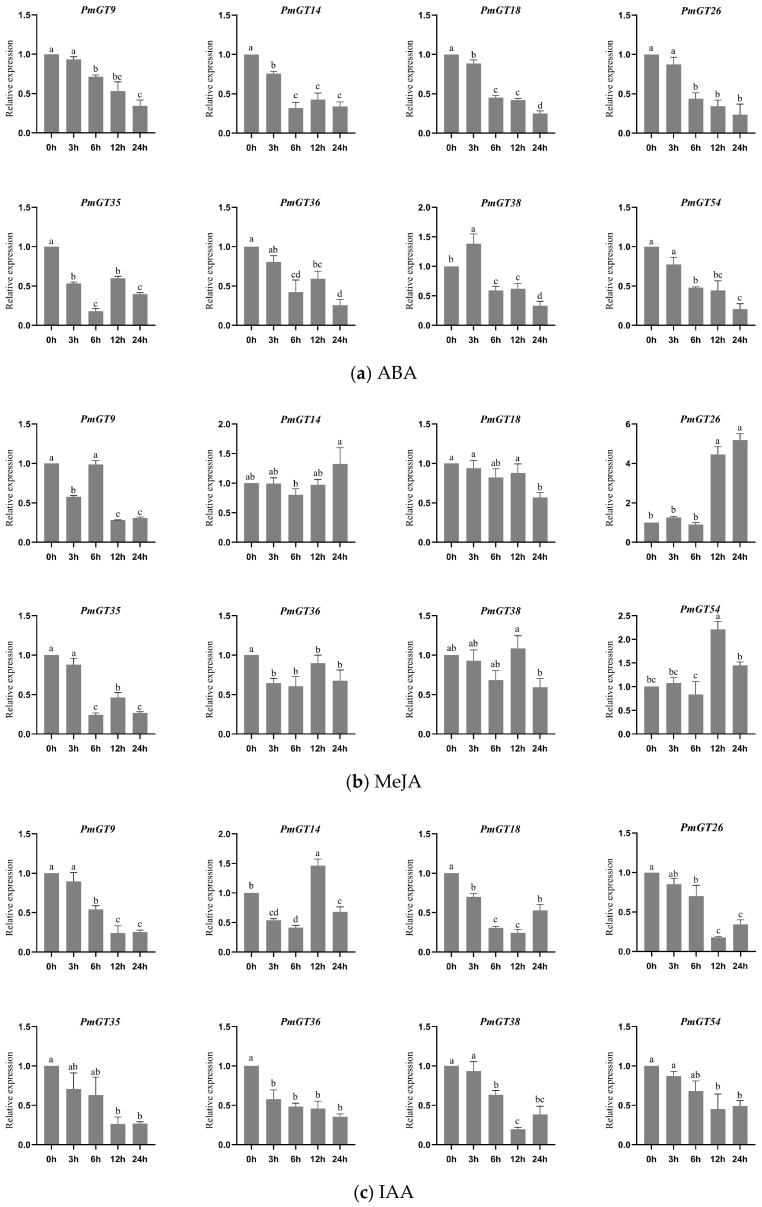

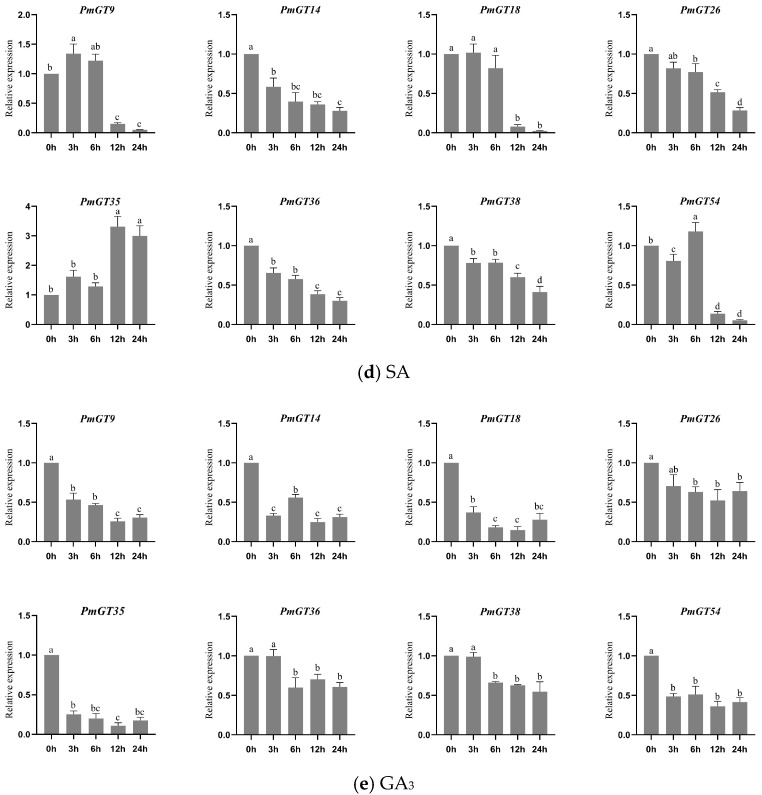
The expression levels of eight *PmGTs* under different treatments. (**a**) ABA, (**b**) MeJA, (**c**) IAA, (**d**) SA, (**e**) GA_3_. Different letters in the same column indicate a significant difference, *p* < 0.05.

Based on the analysis results of the promoter elements, Masson pine seedlings were subjected to different stress treatments (Figure 7). Except for *PmGT38*, the other seven members all showed significant inhibitory effects under ABA treatment, suggesting that under osmotic stress signals, the GT module might be inhibited to reduce energy consumption. *PmGT14*, *PmGT54*, and *PmGT26* can be induced by MeJA, and *PmGT35*, *PmGT9*, *PmGT18*, *PmGT38*, and *PmGT36* were inhibited by MeJA. Under IAA treatment, *PmGT14* showed induced expression at 12 h, and other GT genes were inhibited by IAA. *PmGT35* can be induced by SA after 12 h. Instead, *PmGT14*, *PmGT18*, *PmGT26*, *PmGT38*, and *PmGT36* showed decreased expression under SA treatment. *PmGT9* and *PmGT54* showed increased expression in first 6 h, and decreased significantly after 12 h. All expression of eight GT showed significant inhibition under GA_3_ treatment. Collectively, the hormone expression pattern indicates that the *PmGT* family may act as a signal hub, converting defense-related (JA, SA), growth-related (IAA, GA_3_), and abiotic stress-related (ABA) plant hormones into finely regulated transcriptional outputs, thereby controlling the development and stress adaptation of *P. massoniana*.

### 2.9. Transcriptional Activation

**Figure 8:**
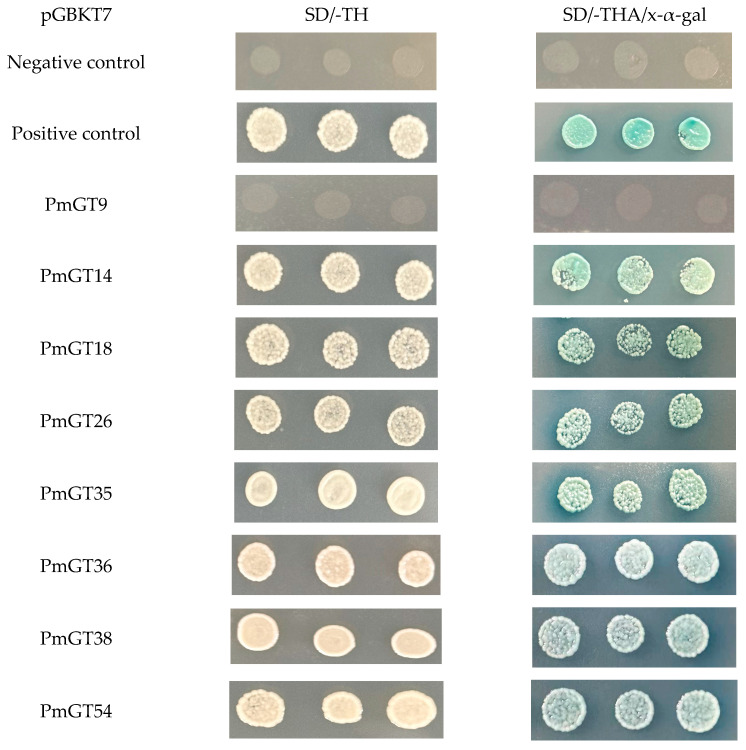
Transcriptional activation assay of eight *PmGT* genes.

TFs can be classified into transcription activation factors and transcription inhibition factors. When activating downstream genes, it is necessary to exclude the influence of their own self-activation capability. Based on the growth images of yeast in the dropout medium, we found that *PmGT9* lacks transcriptional self-activation, while other *PmGTs* exhibit this capability (Figure 8). The yeast transformed with the empty vector pGBKT7 served as the negative control, which cannot grow on either double or triple dropout media. In contrast, the positive control showed that yeast possessing transcriptional autoactivation activity were able to grow on dropout medium. Furthermore, upon addition of the X-α-Gal, Yeast with self-activating will express a reporter gene MEL1, leading to production of α-galactosidase. This enzyme hydrolyzes the colorless X-α-Gal substrate, resulting in the formation of a blue-colored product, thereby verifying functional reporter activation and confirming viable yeast growth under selective conditions.

## 3. Discussion

*P. massoniana* is an economically valuable tree species known for its timber and resin production. Despite its strong vitality and environmental adaptability, it remains highly susceptible to pine wood nematode disease [1]. Both biotic and abiotic stresses severely limit plant growth productivity. To cope with these challenges, plants have developed sophisticated regulatory mechanisms, including transcriptional regulation mediated by TFs, which play a crucial role in coordinating gene expression in response to environmental stimuli [29]. GT genes play a key role in plant morphological development and in responses to external stresses. With the completion of genome sequences for numerous key plant species, systematic identification and genomic-level analysis of GT genes have become feasible across diverse plant species [8,9,30]. Previous studies have highlighted the crucial role of GTs in plant growth and stress tolerance [8]. However, no comprehensive study on GT transcription factors in coniferous species has yet been reported. In this study, we identified the GT gene family in *P. massoniana* and performed a comprehensive analysis to establish a foundation for future research in coniferous trees.

A total of 56 GT genes were identified in *P. massoniana* and mapped across 12 chromosomes (Figure 1A). The number of identified GT genes is about the same as that in *Populus trichocarpa* and *P. tabuliformis* (56 and 59, respectively) [30], but higher than that in *Arabidophsis* and rice (30 and 41, respectively) [8,31], reflecting differences between herbaceous and woody plants. Furthermore, we identified a total of 25 pairs of orthologous GT genes between *P. massoniana* and *P. tabuliformis* (Figure 1B), indicating a close evolutionary relationship and probable common ancestral for these genes. However, gene duplication, mutation and species divergence may be attributed to in the absence of collinearity. Early studies suggested that the GT family genes comprised three distinct subfamilies (GTα, GTβ, and GTγ) [32]. Subsequently, in 2012, Kaplan-Levy et al. proposed a revised classification, redefining the trihelix gene family into five subgroups [8]. In this study, phylogenetic trees indicated that all *PmGTs* were classified into five subgroups, consistent with previous findings (Figure 2A). The conserved domain within the gene is crucial for gene expression and serves as its functional core [33]. Multiple sequence alignment and motif prediction results (Figure 2C,D) indicated that all *PmGT* genes contained a conserved triple helix-loop-helix-loop-helix domain, characteristic of GTs in other plant species. Except for a few genes in each subgroup, most genes exhibited similar gene structures and motif compositions, indicating a close evolutionary relationship.

Promoter cis-regulatory elements are well recognized for their critical roles in regulating plant development and responses to environmental stresses. Analysis of cis-acting elements in the *PmGT* genes promoters revealed that light-responsive elements accounted for more than 50% of all identified elements in *P. massoniana*, suggesting a specialized function in binding to GT elements. Notably, the co-enrichment of TGACG-motif and TCA-element in over 70% of *PmGT* promoters mirrors the jasmonic acid-salicylic acid (JA-SA) antagonistic regulatory module, suggesting that many *PmGT* transcription factors may function as key nodes in the signaling crosstalk during biotic stress responses [26]. In addition, disease resistance associated with these genes is primarily regulated by phytohormone signaling pathways, where SA and JA play central roles in activating plant defense mechanisms [34]. Based on the cis-regulatory element analysis results, we applied several treatments to *P. massoniana* seedlings to investigate the expression patterns of *PmGT* genes under different hormonal conditions.

According to the heatmap results (Figure 4), most *PmGTs* can be induced by PWN, indicating their key roles in PWN resistance. Consistent with these findings, current studies have shown that GT genes are widely involved in plant biotic stress responses. For example, *OsRML1* is induced in response to infection by the rice pathogen *Magnaporthe grisea* [23]. *VvTH08*, *VvTH12*, *VvTH13*, *VvTH15*, and *VvTH22* play crucial roles in anthracnose resistance in *Vitis vinifera* [35]. Based on their strong responsiveness to PWN, eight *PmGTs* were selected for further functional analysis. The subcellular localization results showed that PmGT proteins, similar to *Fragaria vesca*, *A. thaliana*, *O. sativa*, and other plants, are predominantly localized in the nucleus (Figure 5) [36]. Although GT TFs all function within the nucleus, they exhibit diverse functions across species, display tissue-specific roles, and show distinct expression patterns [37]. Gene expression patterns showed that *PmGT* genes are expressed in different tissues, with the highest expression in needles except for *PmGT36* and *PmGT38.* This suggests that most *PmGT* genes may perform specific functions in needles. In contrast, the predominant expression of *PmGT36* and *PmGT38* in female and male cones indicates their specialized roles in the development of Masson pine (Figure 6). Tissue-specific expression patterns indicate their potential functions in multiple aspects of *P. massoniana*.

To investigate the functions of the eight selected *PmGT* genes, their responses to plant hormone treatments were further analyzed (Figure 7). SA and MeJA exhibit cooperative functions in mediating signaling pathways in response to biotic stresses, while ABA plays a broad regulatory role in plant responses to diverse biotic and abiotic challenges. The overexpression of *FvTrihelix6* in *Arabidopsis thaliana* enhanced resistance to *Colletotrichum. higginsianum* by modulating the SA and JA signaling pathways [25]. Similarly, four *PkGT* genes in the SIPI subgroup with more regulatory elements responded and were higher expression after MeJA treatment in *Polygonatum kingianum* [36]. In this study, *PmGT9*, *PmGT35*, and *PmGT54* showed significant responses to SA treatment, while *PmGT14*, *PmGT26*, and *PmGT54* showed significant responses to MeJA, indicating their potential roles in PWN resistance through the SA and JA signaling pathways. Interestingly, all chosen *PmGTs* showed significant inhibition under GA_3_ treatment and ABA treatment. However, *OsGTγ*-1 is upregulated by ABA treatment in rice, highlighting its involvement in stress response pathways [32], and *AtGT2L* can also be induced by salt stress and ABA treatment [37]. These contrasting patterns suggest that GT genes across different plant species may have undergone functional diversification during evolution. Except for *PmGT14*, the remaining seven members showed significant inhibition under IAA treatment. Collectively, these results suggest that *PmGTs* may play a crucial role in hormonal regulation under stress conditions and are likely involved in the defense response against PWN.

Except for *PmGT9*, the other seven members exhibit transcription activation to serve as transcription activation (Figure 8), functioning as either transcriptional activators that promote gene expression or transcriptional repressors that suppress it, depending on their specific roles. In recent years, a growing number of TF families have been identified in *Pinus massoniana* in recent years [38,39,40,41]. However, no research about GTs has been reported to date. Therefore, the identification and comprehensive analysis of *PmGTs* in this study provide a foundation for understanding the function of GT genes in hormonal stress regulation and resistance to PWN in *P.massoniana*.

## 4. Materials and Methods

### 4.1. Identification and Analysis of PmGTs

The hidden Markov model (HMM) profile corresponding to the PmGT domain (PF13837) was obtained from the Pfam database (http://pfam.xfam.org/, accessed 12 March 2024). This HMM profile was then used to identify *PmGT* family members in the genome of *P. massoniana* through a BLASTP search (*p* < 0.001) [42]. A heatmap was generated using TBtools based on transcriptome data from pine wood nematode inoculation (SRA accession: PRJNA660087) [43]. The PmGT proteins were predicted using a TF prediction tool (http://planttfdb.gao-lab.org/prediction.php, accessed 12 March 2024, v5.0). Subsequently, the conserved trihelical domain within the initially identified *PmGTs* was analyzed using Pfam and CDD Search (https://www.ncbi.nlm.nih.gov/Structure/cdd/wrpsb.cgi, accessed 12 March 2024). The phylogenetic analysis of amino acid sequences was conducted using the maximum likelihood (ML) method in MEGA-X, with 1000 bootstrap replicates for statistical support. PmGT protein sequences from *A. thaliana* were retrieved from NCBI. The resulting phylogenetic tree was visualized and annotated using the online platform EvolView (https://www.evolgenius.info/evolview, accessed on 18 March 2024). In order to better determine the distribution of conserved motifs of PmGT proteins, the online program Multiple Expectation Maximization for Motif Elicitation (MEME) (http://meme-suite.org/tools/meme, accessed on 12 March 2024) was used with the number of motifs set to 10. The multiple sequence alignment of PmGT proteins’ conserved domain was conducted in SnapGene (6.0.2) using MUSCLE. Subcellular localization of the PmGT proteins was predicted using CELLO (http://cello.life.nctu.edu.tw/, accessed 12 March 2024) and WoLF PSORT (https://wolfpsort.hgc.jp/, accessed 12 March 2024). The molecular weights and isoelectric points of the identified PmGT proteins were calculated via the ExPASy server (Swiss Institute of Bioinformatics) at https://web.expasy.org/compute_pi/ (accessed 12 March 2024). PlantCARE [44] (https://bioinformatics.psb.ugent.be/webtools/plantcare/html/, accessed 12 March 2024) was employed to analyze the promoter of *PmGTs*. To visualize gene and promoter structure of *PmGT*, chromosome localization and syntenic analysis, TBtools (v2.376) was utilized. The genome sequence and GFF files were utilized to determine the localization of the *PmGT* family. The analysis of syntenic genes within the *P. massoniana* and *P. tabuliformis* GT families was performed using BLAST and MCScanX.

### 4.2. RNA-Seq Data Analysis and Expression Patterns of PmGTs

The highly resistant Masson pine clone ‘Guang 40-5’, planted at the Shahe forest seed breeding center of Anhui Academy of Forestry (118°15′ E, 32°24′ N), was used as plant materials for RNA-seq and tissue-specific expression pattern analysis. Needles from ‘Guang 40-5’ induced with pine wood nematode at different time points (0 d, 3 d, 10 d, 20 d, and 35 d) were collected for RNA-seq analysis (SRA accession: PRJNA660087) Transcript abundance of *PmGT* genes was assessed by calculating Fragments per kilobase per million mapped reads (FPKM). To visualize partial expression profiles, a heat map was constructed based on log2-transformed (FPKM + 1) values using TBtools, with row-wise normalization applied during the analysis. *PmGT* genes that exhibited significant changes in expression levels (q value < 0.05) across at least two of the five time points were identified as differentially expressed genes. Seven representative tissues (needle; male cone; female cone; old stem; young stem; cone and root) were collected for tissue expression patterns analysis. Two-year-old *P. massoniana* seedlings were planted in the pot in the State Key Laboratory of Tree Genetics and Breeding (Nanjing Forestry University) treated with different hormone treatments, the selected seedlings were sprayed independently with 100 µM abscisic acid (ABA); 1 mM SA; 2 mM gibberellic acid (GA_3_); 10 mM methyl jasmonate (MeJA) and 10 mM indole-3-acetic acid (IAA). Afterward, needles were sampled at 0 h, 3 h, 6 h, 12 h, and 24 h after treatment. All samples were collected from three biological replicates.

### 4.3. RNA Extraction and qRT-PCR Analysis

Total RNA was isolated from *P. massoniana* using the FastPure Plant Total RNA Isolation Kit (RC401, Vazyme Biotech, Nanjing, China) according to the manufacturer’s instructions. RNA concentration and purity were determined using a NanoDrop 2000 spectrophotometer (Thermo Fisher Scientific, Waltham, MA, USA), while RNA integrity was assessed via 1% agarose gel electrophoresis. First-strand cDNA synthesis was carried out with the One-step gDNA Removal and cDNA Synthesis Kit (AT311, TransGen Biotech, Beijing, China). Gene-specific primers for quantitative real-time RT-PCR (qRT-PCR) were designed using Primer 5.0 (Table S3). Amplification was performed using SYBR Green Master Mix (11184ES03, Yeasen Biotech, Shanghai, China) on a 10 µL reaction system consisting of 1 µL of 20-fold diluted cDNA, 5 µL of master mix, 0.4 µL of each primer (10 µM), and 3.2 µL of ddH_2_O. The thermal cycling conditions included an initial denaturation at 95 °C for 2 min, followed by 40 cycles of 95 °C for 10 s and 60 °C for 30 s, with the remaining steps following the instrument’s default protocol. Specificity of amplification was confirmed by analyzing melting curves. The alpha-tubulin (TUA) gene served as the internal reference [43]. Each sample included three biological replicates, each with three technical repetitions. Relative gene expression levels were calculated using the 2^−ΔΔCt^ method [∆CT = CT Target − CT TUA. ∆∆Ct = ∆Ct Target − ∆Ct CK]. Statistical significance among groups was evaluated using Duncan’s multiple range test in IBM SPSS Statistics (Version 25), with different lowercase letters indicating significant differences (*p* < 0.05).

### 4.4. Subcellular Localization Analysis

The open reading frames (ORFs) of eight *PmGT* genes, excluding stop codons, were cloned into the pCAMBIA-1302-mGFP5 vector (primers are provided in Table S3). Following PCR confirmation, positive Agrobacterium clones were cultured in LB medium containing kanamycin (50 mg/L) and rifampicin (25 mg/L) until the optical density (OD600) reached 0.6. The bacterial suspension was then mixed with the P19 strain (an RNA silencing suppressor) in infiltration buffer containing 200 µM acetosyringone (AS), 10 mM MgCl_2_, and 10 mM 2-(N-morpholino)ethanesulfonic acid (MES). The resulting mixture was infiltrated into leaves of four-week-old Nicotiana benthamiana plants. After infiltration, the plants were maintained in darkness for 48 h to facilitate protein expression. GFP fluorescence was subsequently visualized using a LSM710 confocal laser scanning microscope (Zeiss, Jena, Germany).

### 4.5. Transcriptional-Activation Activity Assay

To evaluate the transcriptional self-activation potential of *PmGTs*, recombinant plasmids pGBKT7-*PmGTs* were constructed, each harboring the full-length ORF of *PmGT9*, *PmGT14*, *PmGT18*, *PmGT26*, *PmGT35*, *PmGT36*, *PmGT38*, and *PmGT54*. The primers used for cloning are listed in Table S3. The empty pGBKT7 vector served as the negative control, while *PmC3H20*, previously confirmed to exhibit autoactivation [41], was used as the positive control. These constructs were introduced into the yeast strain AH109 (YC1010, Weidi Biotechnology, Shanghai, China) following the standard transformation protocol. Transformed yeasts were selected on SD/-Trp agar plates and incubated at 29 °C for 48 h. Individual colonies were resuspended in 10 µL of ddH_2_O and verified by PCR. Positive clones were further expanded, and the culture was adjusted to 200 µL with ddH_2_O. Then, 5 µL of a diluted suspension was spotted onto SD medium lacking tryptophan, histidine, and adenine (SD/-Trp/-His/-Ade), as well as onto SD/-Trp medium supplemented with X-α-Gal to detect α-galactosidase activity through colorimetric change. Yeast growth and color development were documented by photography.

## 5. Conclusions

In this study, we identified a total of 56 GT genes in *P. massoniana* and conducted a comprehensive analysis of their physicochemical characteristics, cis-acting regulatory elements analysis, chromosomal localization, collinear relationships with *P. tabuliformis*, phylogenetic relationships, expression patterns and transcriptional activation. We selected eight *PmGT* members that exhibit significant responses to PWN stress for further investigation. Expression analyses revealed their potential roles in hormone signaling pathways. They display tissue-specific expression patterns and are localized in the nucleus. Notably, *PmGT35*, *PmGT14*, *PmGT54*, *PmGT18*, *PmGT26*, *PmGT38*, and *PmGT36* demonstrate transcriptional activation activity. These findings not only reveal novel insights into the role of GT TFs in *P. massoniana* but also underscore the potential of these genes for improving Masson pines’ resistance to PWN and abiotic stresses. The next step is to focus on functional validation of their molecular mechanism against PWN stress. This study provided comprehensive information on the *PmGT* gene family and laid the theoretical foundation for understanding the molecular mechanism in stress response.

## Figures and Tables

**Table 1:** Number of conserved structures of GT transcription factor subfamily protein.

Trihelix Subfamily	N-Terminal Trihelix Domain	4th Alpha Helix Domain	Central Alpha Helix Domain	C-Terminal Long α-Helical Domain	Third Amino Acid Residue
GT-1	1	1	0	1	Trp
GT-2	2	2	1	0	C-terminal Trp; N-terminal Phe
SH4	1	0	0	1	Trp
GTγ	1	1	0	1	Phe
SIP1	1	1	0	1	Ile

**Table 2:** Sequences of the 10 motifs of *PmGTs*.

Motif	Length	Motif Consensus	Motif Logo
Motif 1	21	AKQCKDKWENLKKRYKKEKDG	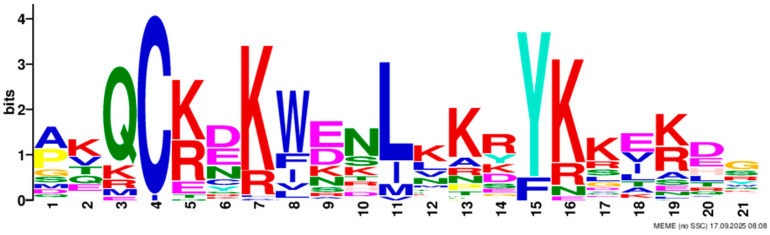
Motif 2	41	ETLALJKIRAEMDSRFRDSKRKKTLWEEISRKLAEKGYRRS	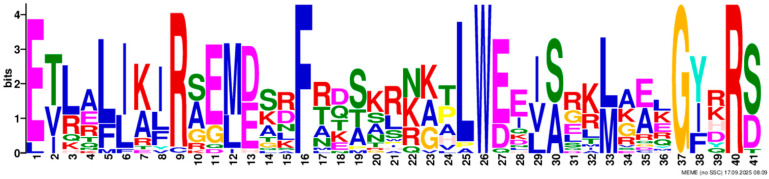
Motif 3	34	QARLEREDZLRAQERALAASRDAAFIALLQKLTG	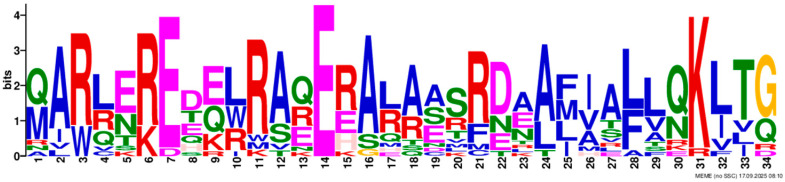
Motif 4	50	DPNSKRWPKPEVLALIRLRSEMEPRFQESGPKGPLWEEISAAMAALGYSR	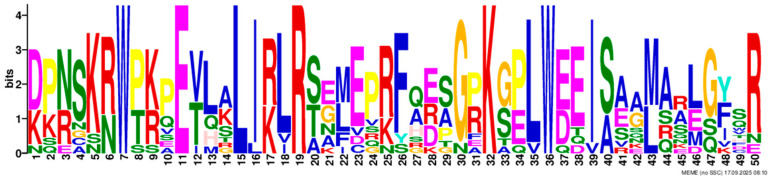
Motif 5	50	TADKKMANFFEELLKQFMZQQERMZQKFLEAIEKREQERMJREEAWKRQE	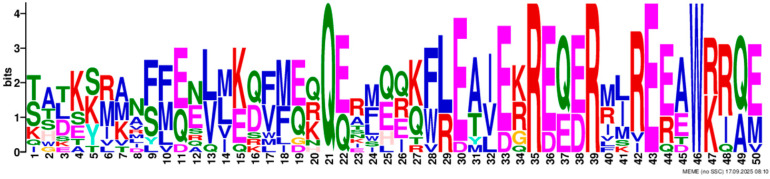
Motif 6	15	SSKWPFFKELDEJLR	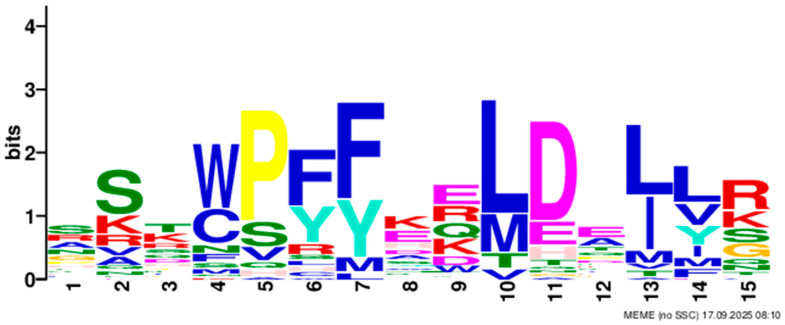
Motif 7	21	NRGNLKKKDWEEVAKAVNARC	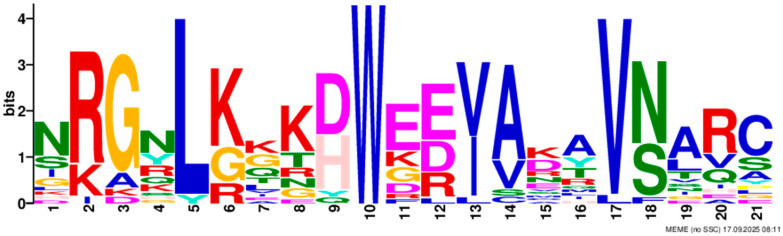
Motif 8	21	RDEWSETAVDTLLDAYEEKCL	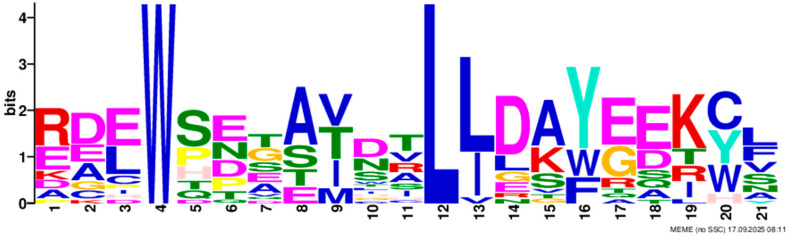
Motif 9	29	NPVRELADAJRSFAEVYERIENAKMEMFK	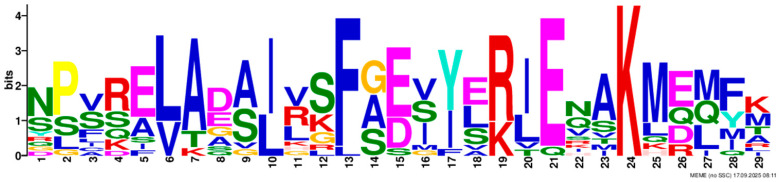
Motif 10	50	NNKMIISNLKMAEEGRMKRHEKYCNLFERRMEMDEKYLNHHAMNVQRMIN	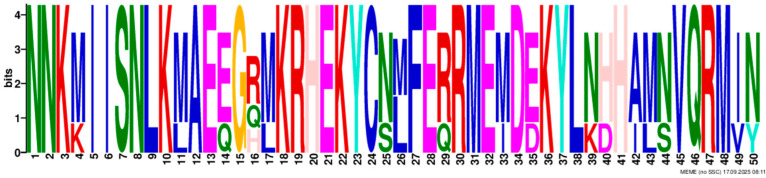

## Data Availability

The original contributions presented in this study are included in the article/Supplementary Material. Further inquiries can be directed to the corresponding author.

## Supplementary Materials

The following supporting information can be downloaded at: https://www.mdpi.com/article/10.3390/plants14233635/s1, Table S1: The characteristics of *PmGTs* and their Subcellular Localization prediction; Table S2: Protein sequences of *PmGTs*; Table S3: The primers used in this study.
